# A Scoring System to Predict the Risk of Postoperative Complications After Laparoscopic Gastrectomy for Gastric Cancer Based on a Large-Scale Retrospective Study

**DOI:** 10.1097/MD.0000000000000812

**Published:** 2015-05-01

**Authors:** Chang-Ming Huang, Ru-Hong Tu, Jian-Xian Lin, Chao-Hui Zheng, Ping Li, Jian-Wei Xie, Jia-Bin Wang, Jun Lu, Qi-Yue Chen, Long-Long Cao, Mi Lin

**Affiliations:** From the Department of Gastric Surgery, Fujian Medical University Union Hospital, Fuzhou, Fujian Province, China.

## Abstract

To investigate the risk factors for postoperative complications following laparoscopic gastrectomy (LG) for gastric cancer and to use the risk factors to develop a predictive scoring system.

Few studies have been designed to develop scoring systems to predict complications after LG for gastric cancer.

We analyzed records of 2170 patients who underwent a LG for gastric cancer. A logistic regression model was used to identify the determinant variables and develop a predictive score.

There were 2170 patients, of whom 299 (13.8%) developed overall complications and 78 (3.6%) developed major complications. A multivariate analysis showed the following adverse risk factors for overall complications: age ≥65 years, body mass index (BMI) ≥ 28 kg/m^2^, tumor with pyloric obstruction, tumor with bleeding, and intraoperative blood loss ≥75 mL; age ≥65 years, a Charlson comorbidity score ≥3, tumor with bleeding and intraoperative blood loss ≥75 mL were identified as independent risk factors for major complications. Based on these factors, the authors developed the following predictive score: low risk (no risk factors), intermediate risk (1 risk factor), and high risk (≥2 risk factors). The overall complication rates were 8.3%, 15.6%, and 29.9% for the low-, intermediate-, and high-risk categories, respectively (*P* < 0.001); the major complication rates in the 3 respective groups were 1.2%, 4.7%, and 10.0% (*P* < 0.001).

This simple scoring system could accurately predict the risk of postoperative complications after LG for gastric cancer. The score might be helpful in the selection of risk-adapted interventions to improve surgical safety.

## INTRODUCTION

Laparoscopic techniques have been used to perform gastrectomies for gastric cancer since they were first reported for early gastric cancer in 1994.^1^ Surgeons have focused closely on the long-term results of laparoscopic gastrectomy (LG) for gastric cancer,^2–4^ and the safety of the procedure has been emphasized. Algorithms of standard treatments, such as the National Comprehensive Cancer Network Clinical Practice Guidelines in Oncology and Japanese Gastric Cancer Treatment Guidelines,^5,6^ have been published to clarify the operative indications. However, because of the differences in the epidemiology of gastric cancer and obesity between Caucasians and Asians, there is no consensus on the difficulty and safety of laparoscopic gastric surgery. Many studies have reported that morbidity rates for laparoscopic surgery range from 11.6% to 18.7%,^7–9^ although some centers have reported rates of 24.9% to 42.6%,^3,4,10^ which hampers the advancement and expanded use of the laparoscopic approach in the treatment of gastric cancer. The identification of patients at high risk for complications might allow the selection of a risk-adapted procedure, and intervening perioperative measures to reduce complications and increase the confidence of the surgeon; therefore, the development of a scoring system to predict the risk of complications is relevant. Few studies have been designed to develop scoring systems that accurately predict the risk of complications.^11–15^ Although the value of a scoring system in predicting complications in patients after an LG has been shown, these scoring systems frequently include many variables and might not be feasibly applicable in clinical practice. In addition, several risk factors have been associated with higher complication rates, such as the age, comorbidities, and body mass index (BMI) of the patient;^9,16–22^ to the best of our knowledge, there is no report of a simple scoring system to predict the risk when multiple risk factors are concurrent. The objective of this study was to identify the risk factors for postoperative complications after laparoscopic radical gastrectomy for gastric cancer in 2170 patients treated in our center. We aimed to use these risk factors to develop a scoring system to predict complications.

## MATERIALS AND METHODS

### Materials

This study was a retrospective analysis of a prospectively collected database of 2170 primary gastric cancer patients treated with a laparoscopic radical gastrectomy in the Department of Gastric Surgery of Fujian Medical University Union Hospital, Fuzhou, China, between May 2007 and December 2013. The patient demographics, underlying diseases, clinicopathology, surgery data, and data on the preoperative and postoperative monitoring were recorded in a clinical data system for gastric cancer surgery.^23^ The staging was performed according to the 7th edition of the International Union against Cancer (UICC) tumor, lymph node, and distant metastasis (TNM) classification.^24^

The inclusion criteria were as follows: a histologically confirmed adenocarcinoma of the stomach; no evidence of tumors invading the adjacent organs (pancreas, spleen, liver, and transverse colon), paraaortic lymph node enlargement, or distant metastasis demonstrated by abdominal computed tomography and/or abdominal ultrasound and posteroanterior chest radiographs; and a D1 + α/D1 + β/D2 lymphadenectomy with curative R0 according to the pathological diagnosis after the operation. The exclusion criteria were as follows: intraoperative evidence of peritoneal dissemination, invasion of the adjacent organs, or a distant metastasis; conversion to an open laparotomy; and incomplete pathological data. The ethics committee of Fujian Union Hospital approved this retrospective study (Approval number: 20070428). All procedures were performed after obtaining written informed consent following an explanation of the surgical and oncological risks. The type of surgical resection (ie, a distal subtotal gastrectomy, proximal subtotal gastrectomy, or total gastrectomy) and the extent of lymph node dissection were selected according to the Japanese gastric cancer treatment guidelines,^6^ as reported in a detailed description in our previous study.^25^

### Variables and Definitions

The definition of each complication was based on the literature.^26–34^ Complications were classified according to the modified version of the Clavien–Dindo classification system reported by Dindo et al.^35^ A grade I complication was defined as any deviation from the normal postoperative course without requirement of pharmacological treatment or surgical, endoscopic, and radiological interventions (with the exceptions of drugs as antiemetics, antipyretics, analgetics, diuretics, electrolytes, or physiotherapy). A grade II complication was defined as any complication that requires pharmacological treatment with drugs other than such allowed for grade I complications (including blood transfusions and total parenteral nutrition). A grade III complication was defined as any complication requiring surgical, endoscopic, or radiological intervention, further subdivided into grades IIIa and IIIb depending on the need for general anesthesia. A grade IV complication was defined as any life-threatening complication (including central nervous system complications) requiring intermediate care/intensive care unit management. Grade IV complications were subdivided into grades IVa and IVb, depending on whether the dysfunction was single- or multi-organ. A grade V complication indicated death of a patient due to a complication. The most severe complication was noted in the cases in which more than one complication occurred in a patient. Complications higher than grade III were defined as “major” complications that are potentially life threatening.^10,35^

The potential risk factors for postoperative complications were extracted from the database, including the sex, age, BMI, previous abdominal surgery, Charlson comorbidity score, hemoglobin (HB) level, albumin (ALB) level, maximum ventilatory volume (MVV), a tumor with pyloric obstruction (diagnosed by gastroscopy or computed tomography scan), tumor with bleeding (hematemesis, melena, or confirmation by gastroscopy), tumor location, tumor diameter, T stage, N stage, TNM stage, operative time (recorded from the skin incision to skin closure), intraoperative blood loss (estimated according to the volume of blood absorbed by the gauze and suction pumped after subtracting the volume of fluids used for irrigation), type of surgical resection, type of reconstruction, D1+/D2 lymphadenectomy, the number of resected lymph nodes, and the operative period (divided into 7 groups).

### Statistical Analysis

The continuous data were reported as the mean ± SD, and the differences between the groups were analyzed using *t* tests. The categorical data were presented as the proportion and percentage and were analyzed with the chi-square test or Fisher's exact test. The variables with *P* < 0.05 in the univariate analysis were subsequently included in a multivariate binary logistic regression model. The variables remaining significant (*P* < 0.05) in the multivariate analysis were used to construct a scoring system to classify the patients into groups according to their risk for complications. A *P* value < 0.05 was considered statistically significant. To assess how well the model could discriminate between patients with and without complications, a receiver operating characteristic (ROC) curve was calculated, and the area under the curve (AUC) was determined, shown as the absolute value and 95% confidence interval (95% CI). The AUC can be interpreted as the probability that a randomly chosen patient with complications will have a higher score than a randomly chosen patient without complications.^36^ The statistical analyses were performed with Statistical Program for Social Sciences (SPSS) version 18.0 (SPSS, Chicago, IL, USA).

## RESULTS

### Clinicopathological Characteristics of the Patients

The clinicopathological characteristics of the 2170 patients are listed in Table 2 . There were 1638 males and 532 females, with a mean age of 61.09 ± 10.75 years. The average BMI of the patients was 22.19 ± 3.07 kg/m^2^. There were 653 patients with a comorbidity (616 patients had a Charlson score of 1–2 points and 37 had a score of 3 points or higher). A total gastrectomy was performed in 1153 patients (53.1%), a distal gastrectomy in 963 patients (44.4%), and a proximal gastrectomy in 54 patients (2.5%); a D1+ lymphadenectomy or D2 lymphadenectomy was performed in 405 patients (18.7%) and 1765 patients (81.3%), respectively. The average surgery time was 180.70 ± 51.54 minutes, including 191.03 ± 50.19 minutes for a total gastrectomy, 169.17 ± 50.95 minutes for a distal gastrectomy, and 153.78 ± 32.80 minutes for a proximal gastrectomy. The blood loss was 73.67 ± 106.95 mL, and the number of dissected lymph nodes per patient was 32.91 ± 12.68. According to the UICC TNM Classification of Malignant Tumors, 7th Edition, 432 patients (19.9%) were in stage Ia, 199 (9.2%) were in stage Ib, 214 (9.9%) were in stage IIa, 247 (11.4%) were in stage IIb, 216 (10.0%) were in stage IIIa, 343 (15.8%) were in stage IIIb, and 519 (23.9%) were in stage IIIc.

### Postoperative Complications

**FIGURE 1:**
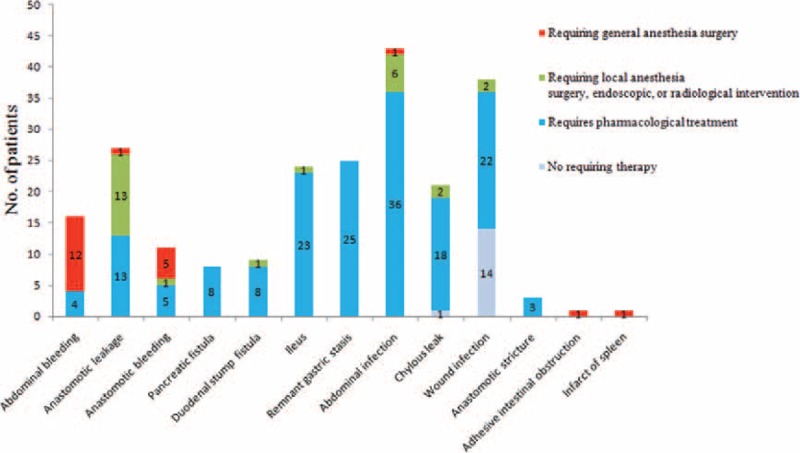
The rates of the local complications and the treatments for the complications.

Table 1 shows the observed morbidities for all of the patients. Postoperative complications were observed in 299 patients (13.8%). Pneumonia (n = 118, 5.4%), intra-abdominal abscess (n = 43, 2.0%), and wound infection (n = 38, 1.8%) were the most common problems among the overall complications. Major complications were observed in 78 patients (3.6%), among which local complications were present in 62.8% of the cases. Severe pneumonia (n = 25, 1.1%), anastomotic leakage (n = 14, 0.6%), and abdominal bleeding (n = 13, 0.6%) requiring surgical, endoscopic, or radiological intervention were the major complications that occurred most frequently. A total of 21 patients required reoperation; the cause was abdominal bleeding in 12 cases, anastomotic bleeding in 5 cases, anastomotic leakage in 1 case, abdominal infection in 1 case, adhesive intestinal obstruction in 1 case, and splenic infarct in 1 case. Figure 1 shows the rates of local complications as well as the treatments for the complications.

**TABLE 1:**
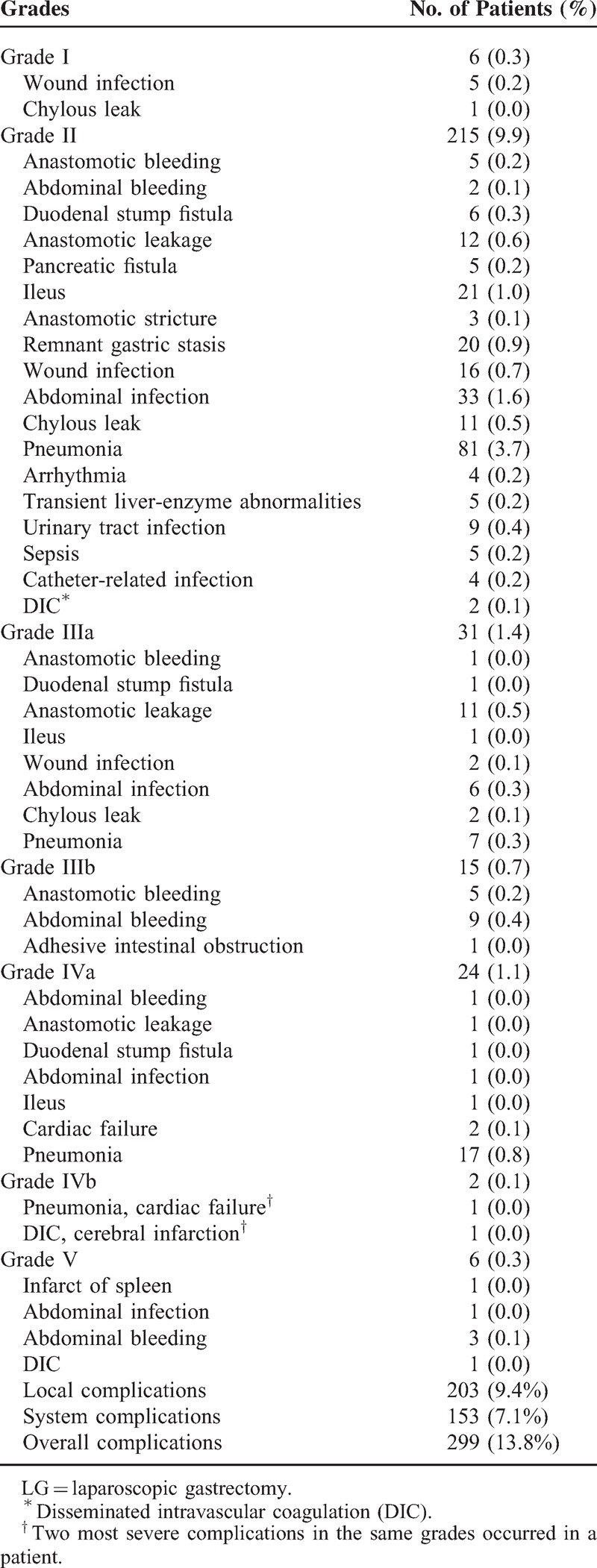
Postoperative Morbidity After LG According to Clavien–Dindo Classification System

Six patients (0.3%) died following the surgery before the 30th postoperative day. The following causes of death were noted, anastomotic leakage and bleeding (2 patients); pancreatic fistula, anastomotic leakage, and bleeding (1 patient); severe pneumonia and abdominal infection (1 patient); splenic infarct (1 patient); and disseminated intravascular coagulation (1 patient).

### Univariable Analyses Associated with Complications

Table 2  shows the results of the univariable analyses of the possible risk factors for the development of complications. Ten factors were associated with an increased risk of overall complications among 22 factors in total: age (*P* < 0.001), the Charlson comorbidity score (*P* = 0.006), BMI (*P* = 0.021), HB level (*P* = 0.031), ALB level (*P* = 0.026), tumor with pyloric obstruction (*P* = 0.001), tumor with bleeding (*P* < 0.001), tumor diameter (*P* = 0.031), intraoperative blood loss (*P* < 0.001), and operative period (*P* = 0.011). Four factors were associated with major complications: age (*P* < 0.001), the Charlson comorbidity score (*P* < 0.001), tumor with bleeding (*P* = 0.002), and intraoperative blood loss (*P* = 0.005).

**TABLE 2:**
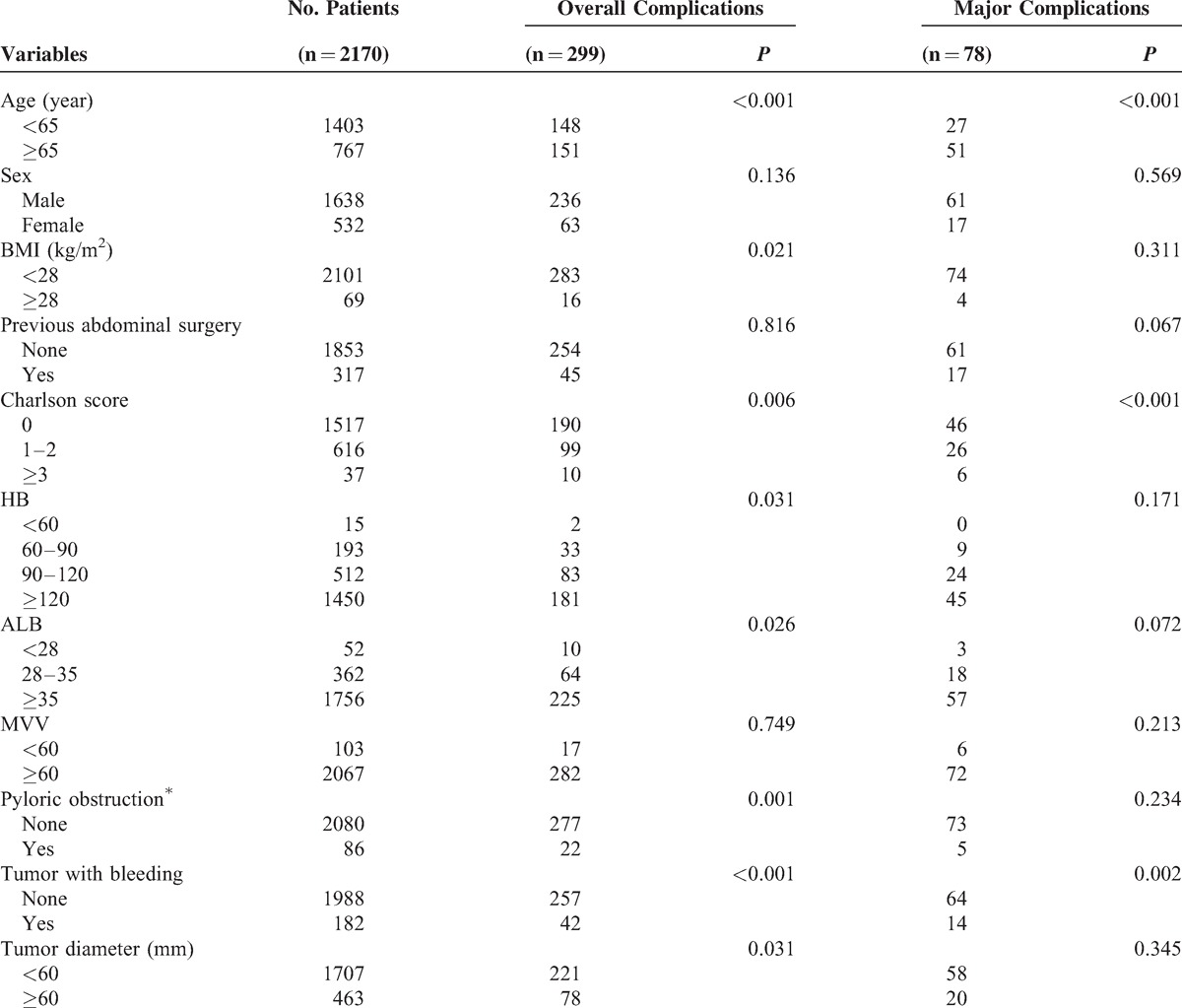
Univariable Analyses of Possible Risk Factors for the Development of Complications

**TABLE 2 (Continued):**
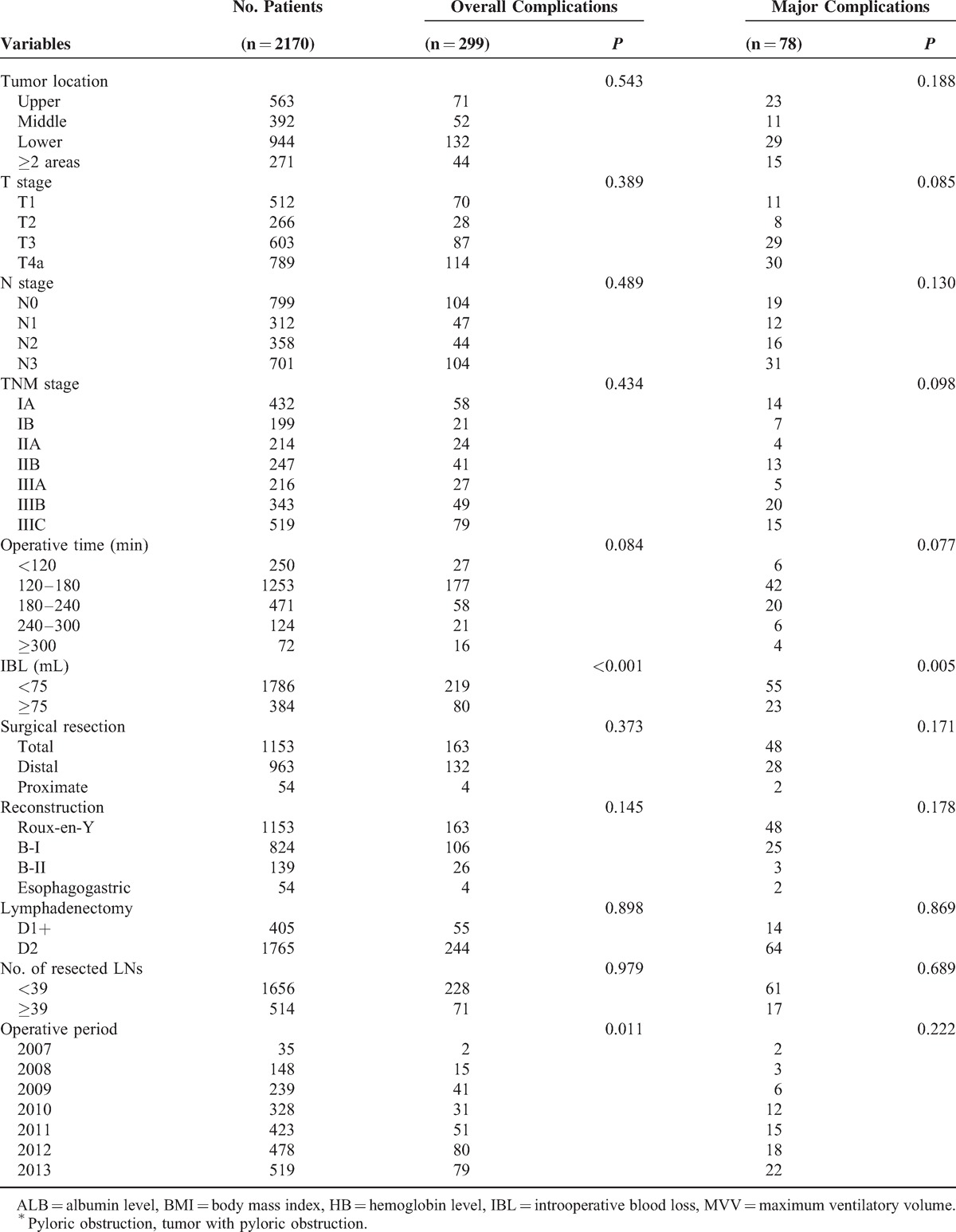
Univariable Analyses of Possible Risk Factors for the Development of Complications

### Multivariate Analysis Associated with Overall Complications and the Scoring System

The multivariate analysis revealed that age ≥65 years [odd ratio (OR) = 2.016, *P* < 0.001], BMI ≥ 28 kg/m^2^ (OR = 1.822, *P* = 0.045), tumor with pyloric obstruction (OR = 2.253, *P* = 0.002), tumor with bleeding (OR = 1.974, *P* < 0.001), and intraoperative blood loss ≥75 mL (OR = 1.797, *P* < 0.001) were independent risk factors for overall complications (Table 3). Despite the differences in the regression coefficients, which ranged from 0.586 to 0.812, for simplicity, 1 point was assigned for each of the risk factors. Because fewer than 5% of the patients had 3 to 5 points, the following 3 risk groups were established low risk (0 points, ie, no risk factors), intermediate risk (1 point, ie, 1 risk factor), and high risk (2–5 points, ie, 2–5 risk factors). The distribution of the patients according to the scoring system was as follows low risk 47.3%, intermediate risk 38.3%, and high risk 14.4%. The incidence rates for overall complications among the patients in the low-, intermediate-, and high-risk categories were 8.3%, 15.6%, and 29.9%, respectively (*P* < 0.001). The relative risk of induction death in the intermediate- and high-risk groups compared with the low-risk category was 2.050 (95% CI, 1.533–2.741, *P* < 0.001) and 4.079 (95% CI, 2.919–5.699, *P* < 0.001), respectively (Table 4).

**TABLE 3:**
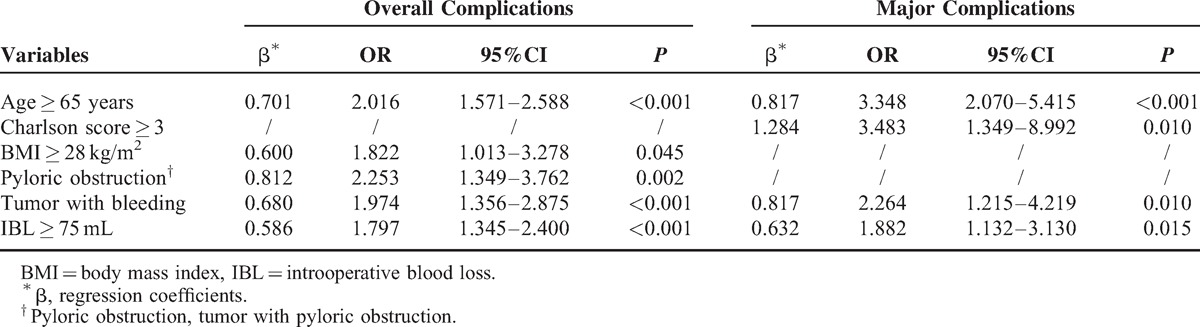
Multivariate Analysis Associated with Complications

**TABLE 4:**
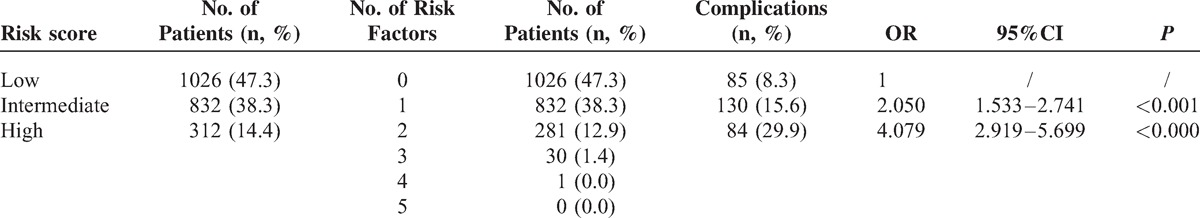
Scoring System for Overall Complications

### Multivariate Analysis Associated with Major Complications and the Scoring System

The multivariate analysis showed that age ≥65 years (OR = 3.348, *P* < 0.001), the Charlson comorbidity score (OR = 3.483, *P* = 0.010), tumor with bleeding (OR = 2.264, *P* = 0.010), and intraoperative blood loss ≥75 mL (OR = 1.882, *P* = 0.015) were independent risk factors for major complications (Table 3). For simplicity, 1 point was assigned for each of these risk factors for which the regression coefficients ranged from 0.632 to 1.284. Because fewer than 5% of the patients had 3 to 4 points, the following 3 risk groups were established low-risk (0 points), intermediate-risk (1 point), and high-risk (2–4 points). The distribution of the patients according to the scoring system was as follows: low-risk 49.9%, intermediate-risk 38.2%, and high-risk 11.9%. The incidence rates of major complications among the patients in the low-, intermediate-, and high-risk categories were 1.2%, 4.7%, and 10.0%, respectively (*P* < 0.001). The relative risk of major complications in the intermediate-risk and high-risk groups compared with the low-risk group was 4.059 (95% CI, 2.153–7.656, *P* < 0.001) and 9.176 (95% CI, 4.646–18.125, *P* < 0.001), respectively (Table 5).

**TABLE 5:**
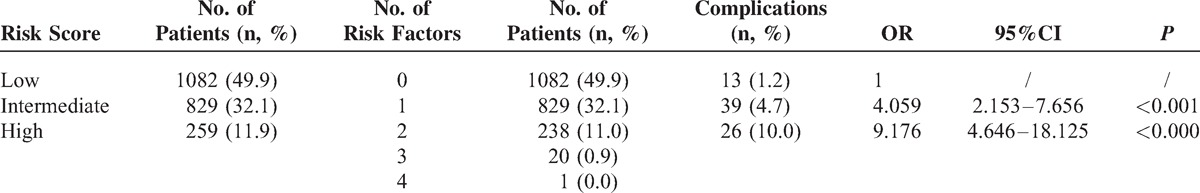
Scoring System for Major Complications

### Discrimination

**FIGURE 2:**
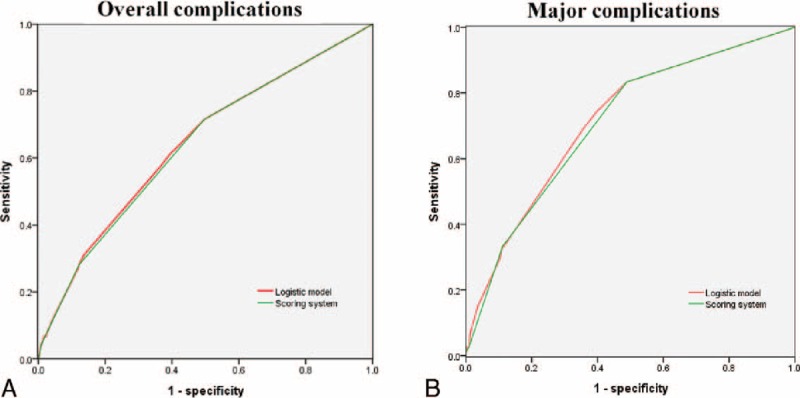
Receiver operating characteristic (ROC) curves for logistic regression model and scoring system predicting (A) overall complications, the area under the ROC curve was 0.641 (0.606–0.675) for the logistic regression model, and 0.637 (0.602–0.671) for the simplified score and (B) major complications, the area under the ROC curve was 0.715 (0.658–0.772) for the logistic regression model, and 0.707 (0.650–0.764) for the simplified score.

The score discriminated between patients with and without complications (overall and major complications) (Tables 4 and 5). The area under the ROC curve was 0.641 (0.606–0.675) for the logistic regression model and 0.637 (0.602–0.671) for the simplified score for overall complications. In addition, the area under the ROC curve was 0.715 (0.658–0.772) for the logistic regression model and 0.707 (0.650–0.764) for the simplified score for major complications (Figure 2).

## DISCUSSION

The development of laparoscopic devices and increased surgical experience has significantly increased the number of laparoscopic surgeries performed in gastric cancer patients. In the literature, reports of laparoscopic D2 lymph node dissections have shown the extent of lymph node dissection and demonstrated that the technical feasibility of the procedures is equivalent to those of open surgery, with no significant difference in the number of resected lymph nodes.^37–39^ Effectively improving LG safety is a global challenge. Surgery safety is subjective, and the incidence of postoperative complications is the most frequently used marker of surgery safety.^10^ Significant differences in the definition and grading of complications have been reported for different surgeons and procedures as well as in surgeries within the same center. Recently, to overcome this problem, surgeons have used the Clavien–Dindo classification system for LG procedures. The system, which was revised and validated in a large cohort of patients who underwent general surgery, has been shown to be an objective and reliable tool for evaluating surgical safety and the severity of complications.^35,40,41^ In reports using this classification system, the rates of overall and major morbidity for laparoscopic surgery vary from 7.0% to 42.6% and 2.1% to 10.6%, respectively.^10,17,42–46^ In this study, the overall and major morbidity rates were 13.8% and 3.6%, respectively. A method of predicting the risk of postoperative complications according to pre- and intra-operative risk factors and appropriate measures to reduce morbidity are needed.

The risk factors associated with postoperative complications after an LG for gastric cancer are controversial. Ryu et al^47^ concluded that the degree of the lymph node dissection and surgical inexperience were risk factors for surgical complications after laparoscopy-assisted distal gastrectomy. Kunisaki et al^48^ reported that there is more surrounding tissue to separate and dissect in patients with a high BMI, particularly in patients with high visceral fat areas; obesity in these patients was associated with significantly higher rates of conversion to open surgery as well as postoperative complications, longer operation times, and greater blood loss. Kim et al^49^ showed that comorbidity, surgical inexperience, proximal reception, older age, and male sex were predictable risk factors for the occurrence of complications. From our data, we found that age ≥65 years, BMI ≥ 28 kg/m^2^, tumor with pyloric obstruction, tumor with bleeding, and intraoperative blood loss ≥75 mL were predictable risk factors for the occurrence of overall complications; age ≥65 years, a Charlson comorbidity score ≥3, tumor with bleeding, and intraoperative blood loss ≥75 mL were identified as independent risk factors for major complications. The patients with one or two comorbidities could frequently tolerate a normal level of surgical stress with preoperative therapeutics and corrections in daily clinical practice. It is difficult to maintain a balance in physiological function with three or more comorbidities, and these patients frequently had major complications. Additionally, our study shows that more attention should be focused on elderly patients, particularly those with other risk factors, despite several recent studies on laparoscopic gastric surgery showing that gastrectomy in the elderly is safe and that older age alone should not be a contraindication to surgery.^17,18^ Our study showed a significantly higher risk of morbidity in patients with preoperative tumor complications, such as pyloric obstruction or tumor with bleeding. This population might be associated with a more advanced tumor stage and poorer nutritional status, which increases the surgical risks and rates of morbidity. In addition, intraoperative blood loss requires additional hemostasis by ligation and compression, and a massive hemorrhage might lead to hypovolemia; these conditions appeared to be associated with poor wound healing and increased infection rates from hypoxia.^50–52^

Although these risk factors were closely related to morbidity, few studies have been designed to create a simple scoring system to predict the risk of morbidity based on multiple risk factors. Previously reported scoring models, such as the Physiologic and Operative Severity Score for the Enumeration of Mortality, the National Surgical Quality Improvement Programme, and the Estimation of Physiologic Ability and Surgical Stress, have been reported to be useful for predicting complications. These scoring modes are not efficacious at the bedside because the models have many required parameters, with 66, 18, and 9 parameters in the National Surgical Quality Improvement Programme, Physiologic and Operative Severity Score for the Enumeration of Mortality, and Estimation of Physiologic Ability and Surgical Stress scoring models, respectively.^11–14^ The Surgical Apgar Score^15^ proposed by Miki uses the following intraoperative parameters: the estimated blood loss, lowest mean arterial pressure, and lowest heart rate. This scoring system is a useful predictor for the development of severe complications, whereas it is useless for selecting risk-adapted preoperative interventions. Our scoring system was based on the final logistic regression model. After giving the same weight to each predictor in the scoring system, the areas under the ROC curves for overall complications and major complications were 0.637 and 0.707, respectively. Both were similar to those in the logistic regression model, which had different weights (overall complications and major complications, 0.641 and 0.715, respectively). Concerning the risk stratification for morbidity, our scoring system classified the patients after LG into 3 groups and identified the highest risk group, which had a 4.1-fold higher risk of overall complications and a 9.2-fold higher risk of major complications than those of the lowest risk group. Patient and disease characteristics data are routinely available, which might have implications for selecting risk-adapted interventions to improve surgical safety. It is impossible to eliminate every risk factor for high-risk patients, such as age; correcting coincident risk factors that may be eliminated or improved by preoperative clinical therapeutics is useful for reducing the morbidity rates. For example, the aggressive treatment of comorbidities, including anemia and malnutrition caused by pyloric obstruction or tumor with bleeding, is required to improve the nutritional status of the patient. Surgical skill is required to identify the vasculature, nerves, and fascia as well as the specific fascial plane to minimize damage to the surrounding tissues and reduce blood loss. Furthermore, it is better to use different procedures for patients with different risks under the rule of complete resection. In addition, care as well as early diagnosis and treatments are necessary to decrease morbidity for high-risk patients. The score was raised in a large series of patients who underwent an LG for gastric cancer. There was a sufficient number of cases in each stage to apply the scoring model in early as well as advanced gastric cancer. Adopting the Clavien–Dindo classification system in our study demonstrated that the score could be easily validated and applied in other centers. The score could be helpful in training physicians in the selection of obvious candidates for laparoscopic surgery, predominantly those with low and intermediate risks of morbidity, which could increase the confidence of surgeons and facilitate progress on the surgery performance learning curve. The score could also facilitate the development of the LG technique for the method to become a universal surgical approach for patients with gastric cancer.

The present study has some limitations. We evaluated patients by age, Charlson comorbidity score, HB level, ALB level, and MVV, but the performance status for some of our cases was not recorded, which might result in some biases. The model shows a good performance for major complications, and the area under the ROC curve for overall complications was approximately 0.65 with a 95% CI of less than 0.61. It might be important to develop a convincing prediction model for overall complications for ordinary patients.

In conclusion, our scoring system allows for the easy risk stratification of morbidity in the clinical setting. This stratification might be helpful for selecting risk-adapted interventions to improve surgical safety. A prospective multiple-center study with a large series would provide valuable evidence for the validation of the score.
